# A Phenomenological and Dynamic View of Homology: Homologs as Persistently Reproducible Modules

**DOI:** 10.1007/s13752-017-0265-7

**Published:** 2017-05-22

**Authors:** Daichi G. Suzuki, Senji Tanaka

**Affiliations:** 1 The Nobel Institute for Neurophysiology, Department of Neuroscience, Karolinska Institute, Stockholm, Sweden; 2 Faculty of Letters, Keio University, Minato, Tokyo, Japan

**Keywords:** Essentialism, Homology, Individuals, Natural kinds, Persistently reproducible modules, PRM view

## Abstract

Homology is a fundamental concept in biology. However, the metaphysical status of homology, especially whether a homolog is a part of an individual or a member of a natural kind, is still a matter of intense debate. The proponents of the individuality view of homology criticize the natural kind view of homology by pointing out that homologs are subject to evolutionary transformation, and natural kinds do not change in the evolutionary process. Conversely, some proponents of the natural kind view of homology argue that a homolog can be construed *both* as a part of an individual *and* a member of a natural kind. They adopt the Homeostatic Property Cluster (HPC) theory of natural kinds, and the theory seems to strongly support their construal. Note that this construal implies the acceptance of essentialism. However, looking back on the history of the concept of homology, we should not overlook the fact that the individuality view was proposed to reject the essentialist interpretation of homology. Moreover, the essentialist notions of natural kinds can, in our view, mislead biologists about the phenomena of homology. Consequently, we need a *non-*essentialist view of homology, which we name the “persistently reproducible module” (PRM) view. This view highlights both the individual-like and kind-like aspects of homologs while stripping down both essentialist and anti-essentialist interpretations of homology. In this article, we articulate the PRM view of homology and explain why it is recommended over the other two views.

Homology, a fundamental concept in biology (Wake 1999; Wagner 2016), provides useful explanations of a broad range of biological phenomena by referring to the historicity of characters (Ereshefsky 2012). However, the concept of homology has been the subject of considerable controversy for a long time (Spemann 1915; Hall 1994, 1999; Laubichler 2000; Wagner 2014). Although there can be no doubt that homology is an important concept in biology, the metaphysical status of homology, especially whether a homolog is a part of an individual or a member of a natural kind, is still a matter of intense debate (cf. Assis and Brigandt 2009; Ereshefsky 2009, 2010b; Wagner 2014).^1^ In particular, the rise of evolutionary developmental biology (EvoDevo) in the past couple of decades has fueled debate over the metaphysical status of homology (see “The Individuality View and the HPC View of Homology” section).

In the following sections, we review the debate between the individuality view and the natural kind view of homology in detail. In the individuality view, homologs are regarded as parts of an individual rather than members of a kind in the metaphysical sense (Ereshefsky 2009, p. 228). In the natural kind view, on the other hand, homologs are regarded as members of a natural kind, an abstract class in the natural world with common essential properties (see the “Homologs as PRMs” section in detail). First, let us identify the point of disagreement. The proponents of the individuality view criticize the natural kind view by pointing out that homologs are subject to evolutionary transformation, and natural kinds do not change in the evolutionary process (e.g., Grant and Kluge 2004). Conversely, some proponents of the natural kind view of homology argue that a homolog can be construed as *both* a part of an individual *and* a member of a natural kind (e.g., Assis and Brigandt 2009; Brigandt 2009). Strictly speaking, they do *not* maintain that the individuality view is entirely wrong. Instead, they emphasize the merits of the pluralistic construal of homologs, which, in their view, will lead biologists to the recognition of novel problems (explananda).

The proponents of the natural kind view adopt the Homeostatic Property Cluster (HPC) theory of natural kinds (cf. Boyd 1999; Wilson 1999). The HPC theory seems to strongly support their construal of a homolog as both a part of an individual and a member of a natural kind. This is because the theory defines a natural kind by using a cluster of properties, which includes historical properties and the very properties that characterize individuals. Here, we are concerned with the validity of this version of the natural kind view of homology, which we call the HPC view of homology below.

When we examine the validity of the natural kind view of homology in general, and the HPC view of homology in particular, we must note that the view includes an essentialist interpretation of homology. If we consider the essentialist claim, the construal of a homolog as both a member of a natural kind and a part of an individual does not make sense. Looking back on the history of the concept of homology, we observe that the individuality view was proposed to reject the essentialist interpretation of homology. The proponents of the natural kind view of homology will reply that they have updated essentialism and that the new kinds of essentialism can fit the individual-like aspects of homologs well. That reply is logically possible but practically futile as the essentialist notions of natural kinds can, in our view, mislead biologists about the phenomena of homology. Alternatively, the individuality view of homology is quite unsatisfactory because it tends to ignore “serial homology.” This is an important aspect of homology, and the concept provides useful explanations in evolutionary developmental biology. Hence, we reject both the essentialist natural kind view and the anti-essentialist individuality view of homology. Instead, we advocate a *non*-essentialist view of homology, which we name the “persistently reproducible module” (PRM) view. This view highlights both the individual-like and kind-like aspects of homologs while stripping down both the essentialist and anti-essentialist interpretations of homology. In a sense, it mediates between the individuality and natural kind views of homology. This article articulates the PRM view of homology and explains why it is better than the other two views.

In the next section, we briefly summarize the history of the concept of homology before the EvoDevo era. In the section following, we review the individuality and natural kind views of homology (especially, the HPC view of homology). Then, in the “Homologs as PRMs” section, we articulate the PRM view of homology and explain its advantage over the two existing views. Finally, in the last section, we briefly explore the possible uses of the PRM view outside biology.

## A Brief History of the Concept of Homology Before the EvoDevo Era

Although it was not called “homology” at the time, the concept of homology can be traced to Aristotle. In *History of Animals*, he distinguished three types of sameness related to biological characters (Aristotle 1965). The first one is specific identity; this type, which would be exemplified by two men with identical noses and eyes, is manifest when two kinds of living thing are specifically identical as a whole (i.e., they belong to the same “species” or *eidos*). The second type is identity with a difference with respect to excess and deficiency. This type of identity is found between two species belonging to the same group (genus or *genos*). In such cases, parts of living things in the same group differ in terms of their secondary characteristics, such as color, shape, and size. The third is pseudo-identity, which is identity by “analogy” or superficial similarity.

In the sixteenth century, Pierre Belon created a famous illustration of homology, providing a comparison of the skeletons of a bird and a man that shows the correspondence of bones (Belon 1555). In the early nineteenth century, comparative anatomists, such as Georges Cuvier and Étienne Geoffroy Saint-Hilaire, analyzed the corresponding structures and organs found in different species in great detail (Russel 1916). Their reports regarded “homology” (it was not yet called that) as the sameness or correspondence of biological characters in different species.

The biologist who first defined homology in a more or less modern way is Richard Owen. In 1843, he clearly distinguished *homology* from *analogy* (Owen 1843).^2^ According to him, homology is not sameness of functions but sameness of characters (organs and structures). He defined a homolog as “the same organ in different animals under every variety of form and function” (1843, p. 379). On the other hand, he defined an analog as “a part or organ in one animal which has the same function as another part or organ in a different animal” (1843, p. 374) In other words, a character is homologous with another because of what it *is* and analogous with another because of what it *does* (cf. de Beer 1971).

Based on this distinction, the paired fins of fish and tetrapod limbs are homologs, whereas the wings of flies, birds, and bats are analogs because they perform the same function (i.e., flying). We now know that these lineages evolved their flying abilities independently of one another and that the sameness of functions is due to the convergent evolution of wings. However, we should note that the wings of birds and bats are homologs *as vertebrate limbs* (birds and bats share the identical vertebrate limb organization derived from their common ancestor). The important point here is the distinction between character identity and character state (cf. Wagner 2007). The vertebrate limbs are homologs and share their character identities, but the character states of the vertebrate limbs are diverse; bird forelimbs possess feathers, whereas bats have parachutes. These diverse structures evolved independently from each other but perform the same function (i.e., flying) in somewhat different ways.

Furthermore, Owen subdivided the homology concept into *special homology* and *general homology* (Owen 1848, pp. 7–8). He defined special homology as the correspondence of parts (or organs) in different animals, and he defined general homology as the higher relationship between a part or a series of parts and the fundamental or general type to which it belongs. In particular, the term *serial homology* is used for a series of general homologs. Today, the use of the term “general homology” is rare, and the term “serial homology” is generally preferred.

**Fig. 1:**
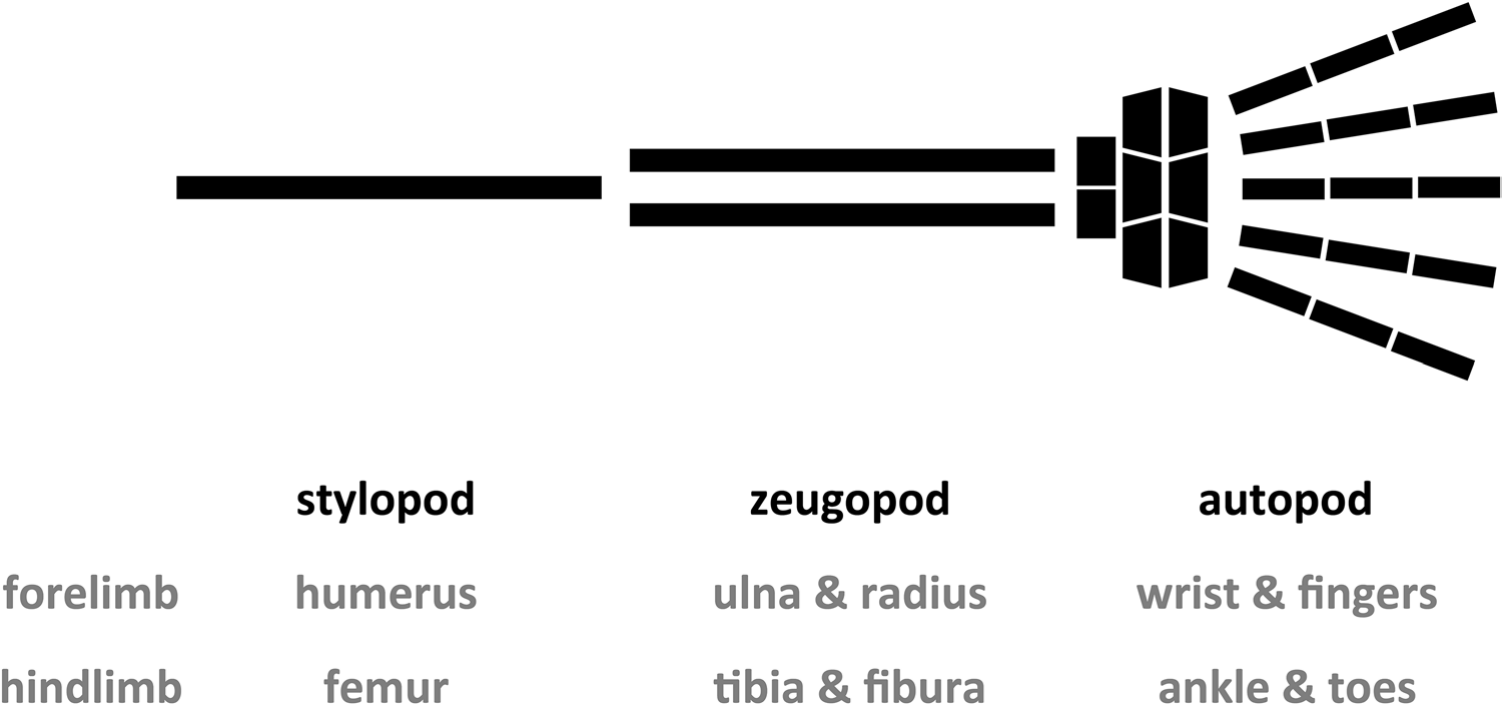
A schematic illustration of the vertebrate limb. Similarity between the tetrapod forelimb and hindlimb has been regarded as a representative example of serial homology, that is, these parts appear to have a general type of osteological structure; the stylopod (the humerus in the forelimb and the femur in the hindlimb), the zeugopod (the ulna and radius in the forelimb, and the tibia and fibula in the hindlimb), and the autopod (the wrist and fingers in the forelimb, and the ankle and toes in the hindlimb)

A representative example of serial homology (or general homology sensu Owen) is that of the tetrapod forelimb and hindlimb. These parts seem to have a general type of osteological structure (Fig. 1). Proximally, there is only one bone; it is the humerus in the forelimb, the femur in the hindlimb, and it is generally called the “stylopod” in the tetrapod limb. Medially, there are two bones; they are the ulna and radius in the forelimb, the tibia and fibula in the hindlimb, and they are generally called the “zeugopod.” The most distal region is generally called the “autopod”; it is the wrist and fingers in the forelimb and the ankle and toes in the hindlimb (Goodrich 1930, p. 159; Wagner 2014, p. 335). There are many other examples of serial homology. One is the segments of arthropods and insects (Snodgrass 1935). Each segment of these animals is thought to have evolved from serial uniform segments, such as those of millipedes (1935, p. 40). Gill slits in vertebrates (Kuratani et al. 2001) and leaves and flowers in plants (Wagner 2014, Chap. 12) are also well-known examples of serial homologs.

Owen’s homology concepts are well known to have been based on essentialism. He thought that homologs share the “essential nature” of animal body parts (Owen 1849, p. 70). On the other hand, Darwin (1859) and his followers considered homology not as the identity with a hypothetical “archetype” but as the signature of common ancestry from the viewpoint of the Darwinian theory of evolution. According to the latter perspective, homologs provide evidence of the affinity between organisms that have evolved from a common ancestor. In particular, Lankester (1870) strongly criticized the essentialist view of homology and pointed out that “no genetic (i.e., phylogenetic or evolutionary) identity can be established between fore and hind limbs” (p. 38), but “the fore legs have a homoplastic agreement with the hind legs” (p. 39). Here, the term “homoplasy” means plastic or ostensible similarity between parts or organs. He also introduced the new term “homogeny” in place of homology to avoid the essentialist connotations of the word “homology,” although this term failed to become popular.

The situation regarding homology is remarkably similar to that of the concept of species. In the second half of the 20th century, it was widely accepted in the fields of biology and philosophy of biology that “the death of essentialism” had occurred with respect to the species problem owing to the Darwinian theory of evolution and phylogenetic systematics (Hull 1965a, b; Ereshefsky 2010a). Instead, the individuality thesis of species became influential (Ghiselin 1974; Hull 1978). The proponents of this thesis argue that species are not natural kinds with an essential nature but are individuals in a metaphysical sense; they emphasize that particular species are defined not by their “essence” but by their history. In the same manner, they criticize the essentialist view of homology and advance the individuality view of homology, which we examine in the next section.

## The Individuality View and the HPC View of Homology

According to the individuality view of homology, this concept is defined as a relationship of correspondence between parts of individual organisms (Ghiselin 2005, p. 97), which are representative individuals in the metaphysical sense. The proponents of the individuality thesis regard homologs as “parts of an individual rather than members of a kind” (Ereshefsky 2009, p. 228).

But, what are individuals in the metaphysical sense? What kinds of entity are they? Let us examine the differences between individuals and natural kinds. According to Ghiselin (1997, 2005), individuals (1) are concrete rather than abstract, (2) engage in process, (3) have no defining properties (i.e., essential properties), (4) have no instances, (5) are spatiotemporally restricted, and (6) do not function in laws. On the other hand, natural kinds (1′) are abstract rather than concrete, (2′) do not engage in process, (3′) have defining properties (i.e., essential properties), (4′) have instances, (5′) are not spatiotemporally restricted, and (6′) function in laws. Thus, there is a sharp contrast between individuals and natural kinds.

“Homology statements are strictly historical propositions,” Ghiselin (2005, p. 95) emphasized; “they are not laws of nature and they lack the necessity that characterizes laws of nature.” Contrary to essentialism, there is nothing like the essential nature of animal body parts that every homolog of animal body parts shares. As Ereshefsky (2009) stresses, homology relationships depend on phylogeny. “…[H]omologs must be historically connected and cannot be spatiotemporally scattered across the universe” (2009, p. 228). Thus, homologs are regarded not as natural kinds but as individuals.

The individuality view seems to fit the Darwinian theory of evolution and phylogenetic systematics well. However, this view tends to ignore serial homology (or “iterative homology”), although the concept of serial homology provides useful explanations in evolutionary developmental biology (de Beer 1971; Roth 1984; Wagner 1989).^3^ In the individuality view, as Lankester (1870) asserted, serial homology is generally explained away as homoplasy (plastic or ostensible similarity between parts or organs).

On the other hand, several contemporary authors (e.g., Rieppel 2005, pp. 25–26; Brigandt 2009, p. 78) embrace the HPC view of homology and argue that homologs are HPC natural kinds. The HPC view is a form of new essentialism, which does not define natural kinds using necessary and sufficient conditions. According to Brigandt (2009), an HPC natural kind has a cluster of properties that permits variation, and there are homeostatic mechanisms that determine the identity of the kind. Here, “homeostasis” means maintenance of the clustering of various properties by underlying causal mechanisms.

It seems that the emergence of the HPC view of homology over the individuality view was accompanied by the rise of EvoDevo (Brigandt 2007, 2009). Many theoretical notions of homology have been proposed in recent decades (e.g., Van Valen 1982 as a precursor; Roth 1984, 1991; Wagner 1989; Abouheif 1997; Shubin et al. 1997, 2009; Müller 2003, 2010; Ochoa and Rasskin-Gutman 2015). These notions basically focus much more on the developmental mechanisms of homologs and criticize “the historical concept of homology” (Wagner 1989; Laubichler 2000), which focuses exclusively on phylogenetic continuity and has a high affinity with the individuality view of homology. Some of these new proposals actually favor the idea of homologs as natural kinds (Wagner 1996, 2014; Rieppel 2005).

The HPC view is different from traditional essentialism, which holds that *every* member of a natural kind has the *same* characteristic, essential properties (cf. Boyd 1999). The HPC view does not require essential properties to be intrinsic or necessary and sufficient for kind membership. Despite the difference, the HPC view is thought to be a kind of essentialism because HPC natural kinds perform the predictive and explanatory roles of traditional essentialist kinds (cf. Wilson et al. 2007; Brigandt 2009).

One critical issue in the HPC view is that the distinctions between individuals and kinds and between natural and functional kinds (hence, the distinction between homology and analogy) becomes vague (Brigandt 2009, p. 77; Wagner 2014, p. 239). For example, proponents of the individuality thesis focus on the historicity of homologs. However, this historicity is easily absorbed in the homeostatic property cluster (not as an intrinsic property but as an extrinsic property) by the HPC view, although the historicity concept has traditionally been connected to the individual concept and disconnected from the kind concept and essentialism. Moreover, the HPC view seems to take scant account of the distinction between natural and functional kinds. The fact that there is no clear-cut distinction between these kinds in general does not rule out that they are two metaphysically distinguishable entities. Proponents of the HPC view attach so much importance to this ambiguity that they tend to conflate different explanatory and classificatory practices in science (cf. Ereshefsky 2009, p. 228).

The same kind of criticism can be applied to another new form of essentialism. According to relational (or historical) essentialism, the essential properties of natural kinds are relational (or historical), and this presumably stands in contrast to their place in traditional essentialism (Griffiths 1999). The relational (or historical) properties are extrinsic ones because (historical) relationships are not intrinsic to members of natural kinds.

In light of relational essentialism, not only species but also individual organisms are natural kinds defined by relations between the individual organism and its parental organisms. Although the idea of “historical essence” might, at first glance, seem to restore essentialism, it spoils the important and evident distinction between two classes of metaphysically distinguishable entities, which have been called individuals and kinds, respectively, in traditional metaphysics. Ereshefsky (2010b) points out that parts of an individual must have certain causal relationships with one another, whereas no such causal requirement is placed on members of a kind. He expresses this idea humorously; “…the tail and the nose of a dog cannot be on different planets and be parts of a single dog: those parts must be causally connected in certain ways” (Ereshefsky 2009, p. 228). On the other hand, members of the paradigmatic kind, such as the element gold, need not be causally connected in any way. At first glance, the new kinds of essentialism seem to fit with scientific practices. However, they actually underestimate the metaphysical diversity of the world. As a result, they lead us into conceptual confusion and provide almost no pragmatic conceptual frameworks for scientific investigation.

As discussed above, the ontology and epistemology of biological phenomena, such as taxa and homologs, are still sources of great controversy (Brigandt 2009; Ereshefsky 2010b). There is a need for a new conceptual framework that is geared to the dynamic aspects of homology and free from conceptual confusion. This situation prompted us to seek an alternative view of homology that can deal with different explanatory and classificatory practices in modern biology better than the individuality view and the HPC view of homology. In the next section, we attempt to provide such an alternative view of homology.

## Homologs as PRMs

In this section, we introduce an alternative view of homology. The distinguishing feature is that it is free from both essentialism and anti-essentialism. It is a *non*-essentialist view of homology. One may be puzzled by this view because it seemingly recommends that one eschew metaphysical investigation of the nature of homology. To elucidate our motivation for a *non*-essentialist view of homology, we want to cite a similar situation in the context of the scientific realism debate.

In 1984, Arthur Fine proposed the “natural ontological attitude” as an alternative position to scientific realism and antirealism. Examining the arguments of the realist and antirealist, Fine found that “*both* the realist *and* the antirealist accept the results of scientific investigation as ‘true,’ on par with more homely truths” (Fine 1984, p. 96; italics added). He calls this acceptance of scientific truths as the “core position” and named it the “natural ontological attitude (NOA).” The NOA is “the core position itself, *and all by itself*” (1984, p. 97; emphasis in original). It is *neither realist nor antirealist* in itself: it mediates between the two. By contrast, each realist and each antirealist makes *additions* to the core position. It is the additions that make each position realist and antirealist and cause them to confront each other. What then are the additions each realist and antirealist makes to the core position? Regarding antirealists, it depends on their specific position. Some antirealists (pragmatists, instrumentalists, or conventionalists) may add to the core position a particular analysis of the concept of truth. Others (idealists, constructivists, phenomenalists, or others) may add a special analysis of concepts or certain methodological strictures. In comparison, realists just add “a desk-thumping, foot-stamping shout of ‘Really!’” (1984). This realist emphasis means to deny the additions that the antirealists make to the core position. Additionally, the realists also want to explain the robust sense of “reality,” which *they assume*. Fine (1984) found that these additions made by each realist and antirealist to the core position were useless and misleading and recommended the core position itself as a third alternative for an adequate philosophical stance toward science.

We do not need to go deep into the scientific realism controversy and argue for NOA here. However, we think that following Fine’s suggestion would lead us to a third alternative to the essentialist natural kind view and the anti-essentialist individuality view of homology. There seems to be a phenomenon of homology that *both* the essentialist *and* the anti-essentialist accept as “true.” In other words, there seems to be a point of agreement between the essentialist and anti-essentialist concerning the phenomena of homology. Let us call this the “core position” of homology. Now, we need to clarify the core position that both the essentialist and anti-essentialist would accept. Following Fine’s lead, we should not make *any* additions to the core position, because they cause useless metaphysical inflation (essentialist or anti-essentialist interpretations).

**Fig. 2:**
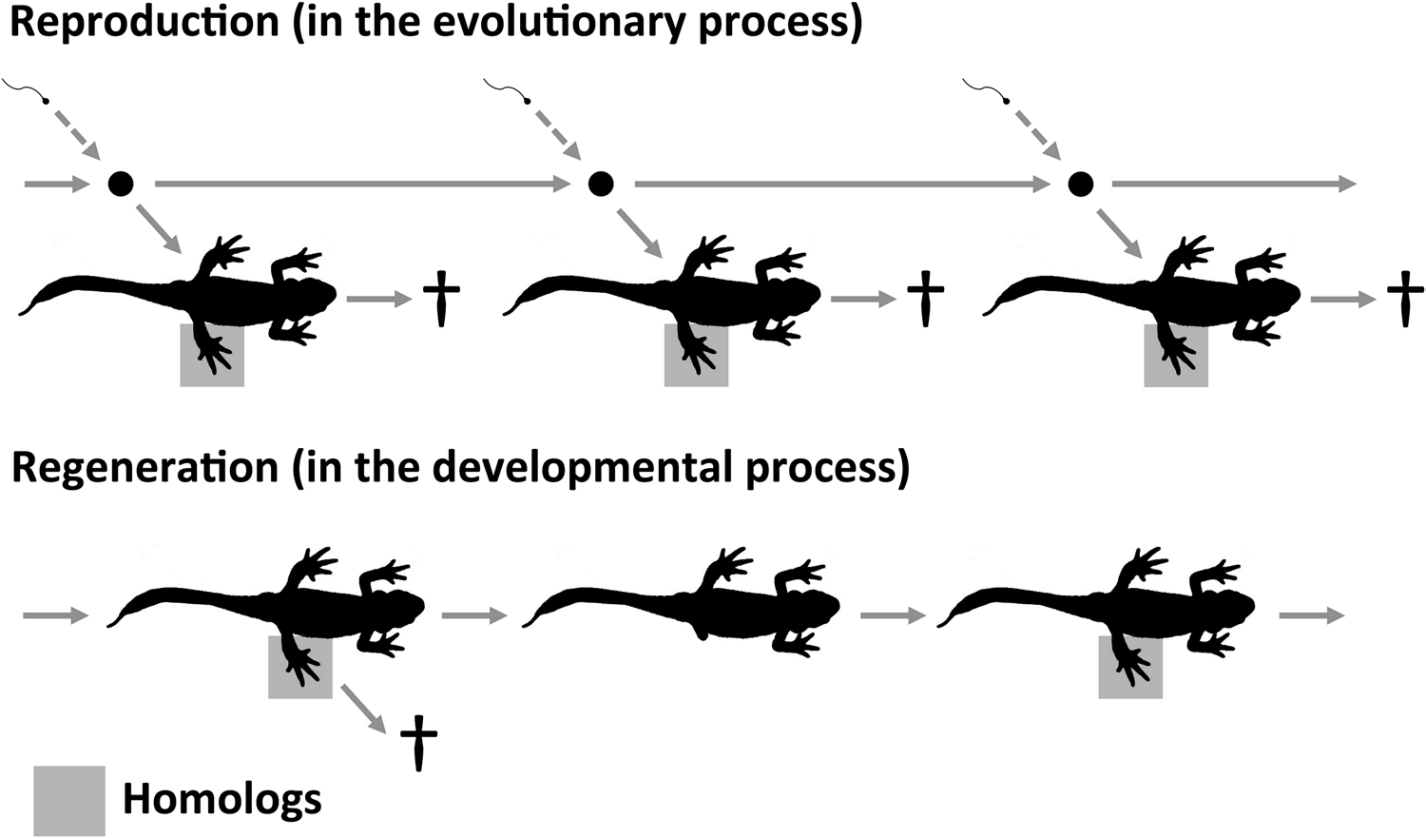
The phenomenology of homologs. In reproduction, which is in the evolutionary process (*above*), homologs form and perish in each generation, although evolutionary lineages maintain their genetic continuity by the inheriting of genetic information. In the developmental process (*below*), homologs are often discontinuous upon regeneration. In both cases, homologs lack continuity and are repetitively generated as autonomous modules

First, we consider that the basic feature of the phenomenon of homology is the *repetitive generation* of homologs (typically, parts of an individual organism, such as limbs or organs) (Fig. 2). This phenomenon is not limited to the evolutionary process (phylogeny), but is also observed in the developmental process. As shown in Fig. 2, homologs are generated repeatedly in each generation via the evolutionary process, as well as in regeneration via the developmental process. Second, we consider the fact that the phenomenon of homology is *autonomous*.^4^ Of course, in the evolutionary process, evolutionary lineages maintain their genetic continuity by the inheritance of genetic information. However, homologs are themselves formed and perish in each generation, and therefore have no genetic continuity (with the exception of asexual reproduction, such as budding). In the developmental process of an individual organism, the same parts are often discontinuous upon regeneration (Fig. 2). There is no continuity between the former part and the newly regenerated one. However, homologs are repeatedly generated in each regeneration via the developmental process. The automaticity of the phenomenon of homology can be partly captured by the concept of modularity. According to Schlosser (2004), modules are integrated, quasi-independent, and autonomous subprocesses.^5^ Using the concept of modularity, we can characterize homologs as *modular* structures distinguishable from other subprocesses in both evolutionary and developmental processes. Focusing on repetitive generation, automaticity, and modularity, we can outline the core position: homologs are *persistently reproducible modules* in evolutionary and developmental processes. Here, we refer to this as the PRM view of homology. Next, we want to articulate the applicability of the PRM view to various evolutionary and developmental processes.

**Fig. 3:**
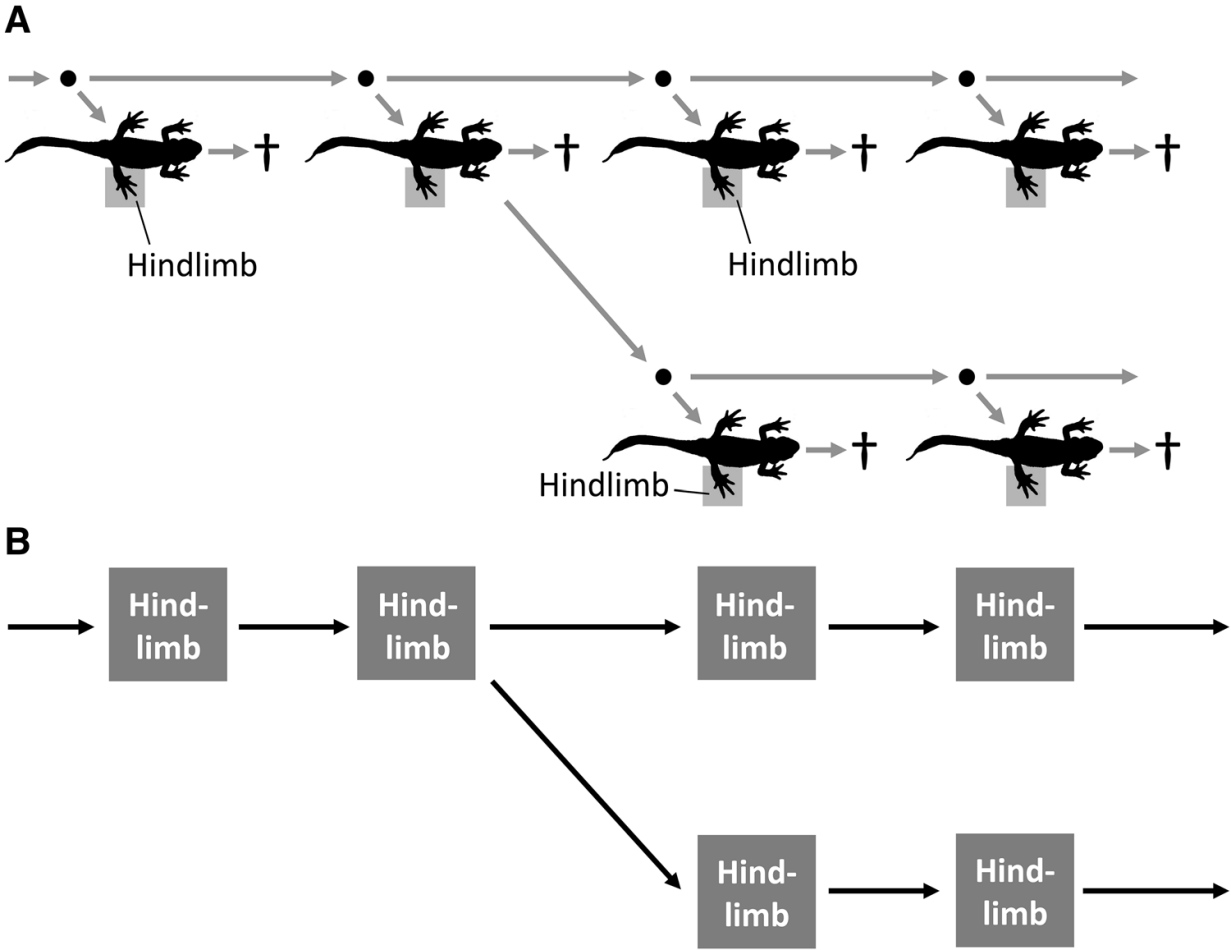
An application of the PRM view in cladogenesis. **a** The cladogenesis of two daughter lineages from a parental lineage. **b** The interpretation of homologs in the cladogenesis in the PRM view. When the cladogenesis occurs, PRMs also split into two daughter PRMs

First, the PRM view can be applied to cladogenesis (Fig. 3). When we observe homologs in two lineages and the lineages are related phylogenetically, the homologs are considered to have been PRMs in the parental lineage. When the parental lineage splits into two (or more) daughter lineages, i.e., a cladogenesis occurs, the PRMs in the parental lineages also split into two PRM lineages. Thus, the characters in the daughter lineages are homologous because they can be traced back to that character in the parental lineage.

**Fig. 4:**
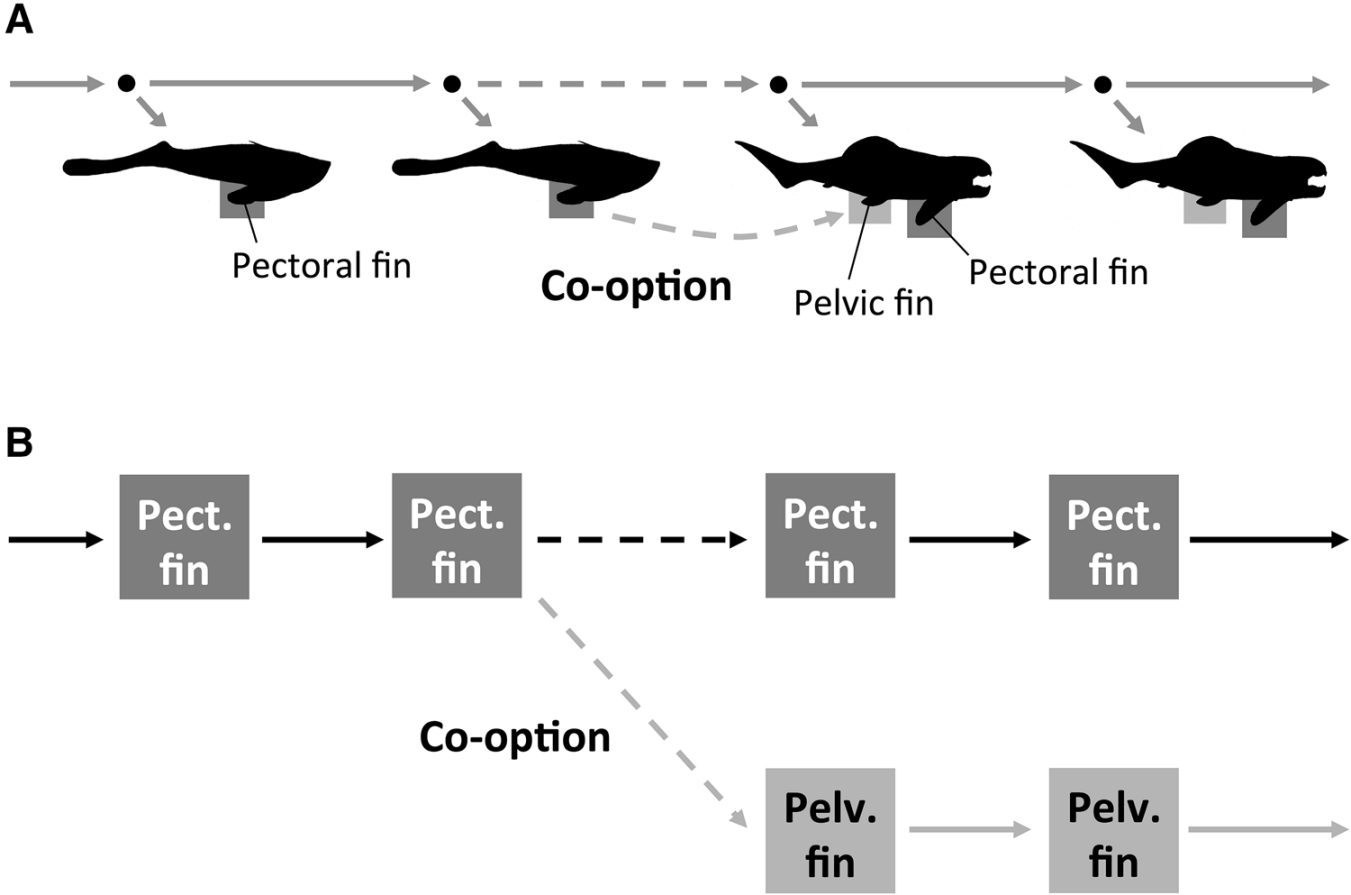
An application of the PRM view in serial homology. **a** The evolution of vertebrate paired appendages. The pelvic fin is thought to have evolved by co-option of a developmental mechanism to form the pectoral fin. **b** The interpretation of serial homology in the PRM view. Note that serial homology can be treated as similar to the case of cladogenesis depicted in Fig. 3

It is worth noting that the PRM view is also applicable to serial homology. For example, in the evolution of the vertebrate paired appendages, the pectoral appendage first appeared and the pelvic appendage subsequently evolved (Young 2010). The co-option of a developmental mechanism (originating from that of the midline fin) seems to have participated in this process (Shubin et al. 1997; Feritas et al. 2006; Shimeld and Donoghue 2012) (Fig. 4a). Based on the PRM view, serial homology can be treated as similar to the case of cladogenesis mentioned above (Fig. 4b). This suggests a unifying framework for the ontogeny and phylogeny of corresponding characters.

There is actually a clear difference between the evolution of special homology and that of serial homology. This difference is comparable to the difference between the evolution of orthologous genes and that of paralogous genes, as Wagner (2014) pointed out. In the case of the evolution of serial homology, one should pay attention to the level of modules in the developmental hierarchy. In the evolution of vertebrate paired appendages, the developmental mechanisms within fore- and hindlimb buds is highly conserved, although this is less the case for characteristics at later stages, such as muscle structures (Diogo and Ziermann 2015). However, this is not only a specific case in serial homology, but it is also observed in special homology. For example, as mentioned above, the wings of birds and bats are homologous at the limb level, but they possess non-homologous structures (feathers and a parachute, respectively) for the flying function.

In both cases, homologs as PRMs permit *modest* generalizations because we can assume some basal mechanisms behind the persistent reproducibility of homologs by comparing homologs between species (in the case of special homology) or organs (in the case of serial homology). However, contrasting the HPC view of homology, the PRM view does not *necessarily* require basal mechanisms to be *essential*. In other words, the PRM view denies that there are basal mechanisms that enable *robust* generalizations. Indeed, developmental mechanisms often diverge over time without accompanying changes in the phenotypic outcomes. This phenomenon is known as developmental system drift (DSD) (True and Haag 2001). In philosophical jargon, this situation is called “multiple realizability”; homologs are multiply realizable at the phenotypic level and can be realized by many distinct developmental mechanisms or cannot be reduced to a single set of developmental mechanisms (cf. Ereshefsky 2012, p. 394). Again, we emphasize the automaticity of the phenomenon of homology: homologs can be generated repetitively even if the basal underlying mechanisms are subject to profound change and variation.

The neurulation process in vertebrates is a notable example of DSD. In amniotes (e.g., *Xenopus*), inhibition of bone morphogenetic protein (BMP) family signaling molecules is necessary and sufficient to influence neural fates. However, in amniotes (e.g., chicks), inhibition of the BMP pathway causes no obvious defects in neural specification, suggesting that some other factors replace or function redundantly with BMP signaling to specify the neural plate. Thus, the PRM view of homology can adequately explain a dynamic aspect of homology; the phenomenon of the multiple realizability of homologs suggests that homology undergoes dynamic changes during the evolutionary process.^6^ We should pay attention to this aspect and be careful not to make excessive generalizations.

Let us show some advantages of the PRM view of homology over the two existing views by examining the color patterns of colored carp (Koi). A variety of colored carp, called *Kohaku* (red–white), exhibits a red–white color pattern (Axelrod 1988). In this variety, the color pattern of the trunk varies, whereas spots on the head are often observed. There is actually a further modified variety, which shows a red spot only at the top of the head. This variety is called *Tancho* (red-cap). In this variety, it is possible to identify the head spots as homologs (a shared derived character; i.e., synapomorphy) of the body color pattern in this variety, that is, this evolutionary lineage. For the head spots to be identified as homologs, they must be modules, because homologs are modules in the PRM view. The head spots are regarded as modules when they are observed only at the top of the head, or at least when they are separated from other trunk spots. When the head spots are repeatedly observed in the lineage and recognized as modules, they are PRMs according to the PRM view.

If we regard the head spots of the variety *Tancho* as PRMs, we can predict that there are genetically fixed developmental mechanisms for the generation of the head spots. Interestingly, we can find similar varieties of goldfish that show a red spot only at the top of the head (Matsui 1972). It would be interesting to investigate the developmental mechanism behind the pattern observed in each variety and to examine the diversity and commonality of the mechanism. Note that this question is more consistent with the PRM view than with the HPC view, because the HPC view would impatiently tend to find “deep homology” between two lineages and asks whether the common basal mechanisms are conserved between colored carp and goldfish (the case for deep homology is discussed in detail below). However, the head spots of the two lineages are actually the results of convergent evolution (Wang and Li 2004; Komiyama et al. 2009). We should avoid prematurely deciding that there are some common developmental mechanisms, such as deep homology, because the existence of such mechanisms depends on species or lineages, and the PRM view can avoid such premature and broad generalizations. In the PRM view, the head spots of the two lineages are regarded as distinct (non-homologous) PRM lineages. Consequently, the PRM view of homology requires a more temperate methodology of evolutionary developmental biology (EvoDevo). The PRM view warns of the risk of assuming “essential” properties behind the phenomena of homology a priori and supports instead the extraordinary diversity of nature.

Notably, the PRM view of homology can incorporate several advantages of the individuality view and the HPC view. In other words, the PRM view can accommodate both the individuality view and the HPC view.

First, the PRM view of homology highlights the important fact that homologs are historical (spatiotemporally restricted) entities engaging in evolutionary or developmental processes. Suppose that a series of modules start to reproduce persistently at some point of time: when this persistent reproduction ends, the series ceases, and the homologs become extinct. In the example of fish coloration, the persistent reproduction of the head spot in colored carp starts independently from that in goldfish varieties. Even if the head spots in both varieties are quite similar, and they may share many properties, they are not homologs because they are the result of convergent evolution in each variety. The head spots in each variety are PRMs with a fate of their own—they engage in the evolutionary processes as something like individuals. Therefore, the PRM view enables more accurate recognition of the phenomenon of homology than the natural kind view.

Second, the PRM view can attribute predictive and explanatory roles to the PRMs in biological investigations. In a sense, the PRMs have *somewhat* kind-like roles. However, we must draw attention to the difference between natural kinds and PRMs. The natural kind view typically emphasizes the predictive and explanatory roles of “essence,” which are assumed to underlie the natural kind (cf. Wilson et al. 2007; Brigandt 2009). By contrast, the predictive and explanatory roles that the PRM view attributes to PRMs in biological investigations are relatively modest ones. As we have already discussed above, the PRM view can warn of the risk of assuming an “essential” spot-forming mechanism behind the head spots of the varieties of both carp and goldfish a priori, although it is scientifically interesting to examine the diversity and commonality of the spot-forming mechanisms between these two lineages.

We noted that the HPC view tends to find deep homology between lineages. The term “deep homology” refers to sharing the same genetic regulatory apparatus that is used to build morphologically and phylogenetically disparate (i.e., non-homologous) characters (Shubin 1997, 2009). For example, the *Drosophila melanogaster* gene *Distal-less* (*Dll*) and its mouse homolog *Dlx* control appendage development in each animal, even though these appendages are not homologous (i.e., the appendages of insects and vertebrates evolved independently in these two lineages). However, they are homologous at the “deeper” GRN level.

The deep homology concept has strong affinity to the HPC view because the “deeper” GRN can be regarded as a basal mechanism leading to homeostasis. Recall that basal mechanisms underlying homeostatic properties play an essential role in the HPC view. In contrast, the PRM view focuses on the *phenomenological* level rather than the basal-mechanism level of homology. As the appendages of insects and vertebrates evolved independently in these two lineages, they are not homologous at the phenomenological level, even if at first glance the shared GRN suggests homology at the deeper level. If there is a basal mechanism for vertebrate limb development as a somewhat conserved GRN and if this GRN is also conserved in *Drosophila* as deep homology, what is the difference between the basal mechanisms *for vertebrate appendages* (which are, in fact, homologous as appendages) and those *for Drosophila and vertebrates appendages* (which are not homologous as appendages)? They are hardly distinguishable! As such, there should be no clear boundary between homology and deep homology in the HPC view.

Atavisms are another example that sheds light on the PRM view. For example, some sperm whales have been reported to have visible hind legs (Berzin 1972). In this case, an interesting issue is why and how the persistent reproducibility of hind legs was once lost in ancestral whales but has reappeared in the current lineage. In fact, all whale embryos possess limb buds at some period of development, but these generally disappear before cartilage formation (Hall 1984). Against this background, it can be considered that the developmental modules of hind legs retain persistent reproducibility at least at the limb bud level, even though they remain lost at the mature hind leg level. Thus, the PRM concept can be applied to various levels of biological processes; that is, not only mature phenotypes but also developmental modules are candidates for PRMs.

In summary, taking the phenomenology of homology seriously, we regard homologs as PRMs in both evolutionary and developmental processes. PRMs are not only restricted spatiotemporally but can also be used to make modest biological generalizations. Based on these generalizations, the PRM view can play predictive and explanatory roles in scientific investigations. Furthermore, this view can accommodate the fact that homologs are subject to dynamic evolutionary and developmental changes. Why is the PRM view preferable to the individuality and natural kind views? It is because the PRM view is the “core position” to which the proponents of the other two views can admit, and it makes no additions that cause useless metaphysical inflation (i.e., essentialist or anti-essentialist interpretations).

## The Scope and Perspective of the PRM View

It is worth noting that the PRM view can be applied to various phenomena outside biology. In this section, we discuss the intriguing applicability of PRMs to diverse natural phenomena.

First, the PRM view can also be applied to species; species are groups of persistently reproducible modules, with these modules being what we usually call individual organisms. Individual organisms themselves are PRMs because they are persistently reproducible, somewhat integrated, quasi-independent, and autonomous subprocesses in evolutionary processes (which are usually called species, evolutionary lineages, or populations).

One may notice that the PRM view has the potential to be applied to other kinds of natural phenomena, including behavioral and psychological phenomena. In fact, some authors have attempted to apply the concept of homology to these phenomena (Lorenz 1958, 1973; Love 2007; Hall 2013; Brown 2014).^7^ For example, some courtship behaviors or emotions can be regarded as homologs. The PRM view seems to be applicable to these phenomena because behavioral and psychological phenomena have modular structures and are persistently reproducible in evolutionary and developmental processes. As for courtship behavior, it is persistently reproduced in the evolutionary process (it should be conserved in the species) and during the life cycle of individuals (an individual may show such behavior many times over the course of its lifetime).

Thus, the PRM view can provide a new viewpoint for understanding the metaphysically diverse natural world and an adequate conceptual framework for scientific investigations. However, when applying this view to appropriate phenomena, careful examination is needed in terms of what modules are and how (much) they are persistently reproduced. Through this examination, the application of the PRM view to various research fields would set a new research agenda and provide a useful perspective.

In this article, we propose a new view of homology, the PRM view, to provide a non-essentialist standpoint as the “core position” in Fine’s (1984) sense, stripping down both the essentialist and anti-essentialist interpretations of homology. Actually this view has affinity with other recent homology concepts that have been proposed from the developmental perspective of homology,^8^ indicating the adequacy of this view for biologists’ daily use. By emphasizing the basic features of the phenomenon of homology, this view regards homologs as PRMs in evolutionary and developmental processes. PRMs are not only spatiotemporally restricted but can also be used to make biological generalizations. Based on these generalizations, the PRM view can perform predictive and explanatory roles in scientific investigations. Moreover, this view can accommodate the fact that homologs can change dynamically in evolutionary and developmental processes. It can also be applied to various domains outside biology, such as those involving behavioral and psychological phenomena.

## Footnotes

1 This situation is strikingly similar to that of the species problem (Ereshefsky 2007, 2010a).

2 According to Panchen (1994), Owen was not the first biologist to introduce this distinction. William Sharp MacLeay had already made this distinction in 1821, and Owen reinforced it.

3 “Iterative homology is ‘a misnomer’, according to the proponents of the historical homology concept, ‘because it is not concerned with tracing organs in different organisms to their representatives in a common ancestor’ [de Beer 1971, p. 9]” (Wagner 1989, p. 54). We should take it seriously that “serial and other forms of iterative homology have been [...] overlooked” (Roth 1984, p. 13).

4 The importance of autonomy for homology was also emphasized in previous works (e.g., Wagner 1989; Müller and Newman 1999; Schlosser 2004). In addition, the lack of continuity among homologs was noted previously by Wagner (1989, 2014).

5 Modular systems abound in our world, especially in biological phenomena (Schlosser and Wagner 2004; Callebaut and Rasskin-Gutman 2005). Morphological modules, to which we refer in this article, can also be studied and characterized differently in a variety of biological contexts and experimental or observational settings: developmental, genetic, functional, and evolutionary modules (reviewed in Klingenberg 2008). In evolutionary processes, these morphological modules can be both conserved (e.g., Koyabu et al. 2014) and rearranged (e.g., Suzuki 2013; Suzuki et al. 2014).

6 Here we refer to “dynamic” to emphasize that the characteristics of homologs can be spatiotemporally changed in biological processes, in contrast to the static (“homeo-*static*”) perspective of the natural kinds views, such as the HPC view.

7 As Lorenz (1958, 1973) noted, the idea of behavioral homology can be further traced back to Charles Otis Whitman and Oskar Heinroth. “Instincts and organs are to be studied from the common viewpoint of phyletic descent” (Whitman, quoted in Lorenz 1958, p. 69). See also Podos’s (1994) historical review of the ideas of Whitman and Heinroth and the literature cited within.

8 The PRM view appears to be similar especially to the organizational homology concept of Müller (2003). However, given that this concept focuses much more on the roles of homologs in organizing phenotypes and “evoloving molecular and genetic circuitries,” it differs from our PRM view.
